# The Effects of the COVID-19 Pandemic on the Pattern of Fractures in Taif City, Saudi Arabia

**DOI:** 10.7759/cureus.57680

**Published:** 2024-04-05

**Authors:** Ahmed Alzeyadi, Renad A Alqahtani, Lara E Alsulimany, Rahaf M Alsudani, Areej A Turkistani

**Affiliations:** 1 Orthopaedic Surgery, King Abdulaziz Specialist Hospital, Taif, SAU; 2 Medicine, Taif University, Taif, SAU; 3 Pharmacology and Toxicology, Taif University, Taif, SAU

**Keywords:** sars-cov-2, emergency, pattern of fractures, fractures, pandemic, covid-19

## Abstract

Background: The COVID-19 pandemic forced the world to take restricted orders to control the spread of SARS-CoV-2 by limiting and controlling people's movement inside their countries. According to previous studies, the shutdown and quarantine affected orthopedic trauma presentations and patterns in many countries. This study aimed to show the change in the pattern of fractures in Taif City, Saudi Arabia, during the curfew period during the COVID-19 pandemic in 2020 and compare it with the same period in 2019.

Methods: A retrospective study of all patients with fractures who came to the emergency room and were treated by the orthopedic department at Alhada Armed Forces Hospital, King Abdulaziz Specialist Hospital, and King Faisal Medical Complex, Taif City. During partial and total lockdowns between March 23 and June 21, 2020, Data was collected from this period during September 2023. Demographics, fracture type and site, mechanism of injury, length of admission, if admitted, and the management plan were documented. These fracture cases were compared with those within the same period of March-June from the previous year. All statistical analysis was done using two-tailed tests.

Results: There was a decrease in the number of fractures at the time of quarantine, with 69 cases in the three months of curfew in 2020, compared to 184 cases in the same three months in 2019. There was a significant difference in the anatomical site of fractures between the two years, with decreased radius/ulna fractures (P= 0.035) and foot fractures (P=0.010). Most fractures during the lockdown were due to home accidents, with a significant decrease in motor vehicle accidents (P=0.009).

Conclusion: The pattern of fractures in Taif City was affected by the movement restriction during the COVID-19 pandemic. The number of fractures decreased by 62.5% during the quarantine compared with the pre-pandemic period. A significant decrease was noted in the number of hospitalized patients (33%) during the COVID-19 lockdown compared to 65.8% in the pre-pandemic period.

## Introduction

Coronavirus disease (COVID-19) is an infectious disease caused by the SARS-CoV-2 virus [1]. Most people infected with the virus suffered from mild to moderate respiratory illness, and the symptoms often started with fever, cough, fatigue, and myalgia. In some patients, there was loss of taste or smell. The virus can spread easily from infected people to healthy people through respiratory droplets when coughing or sneezing [1]. On March 11, 2020, the WHO announced COVID-19 as a pandemic [1]. From this point, many countries implemented curfews and quarantine as a defense plan to limit the spread of the virus.

After confirming the first case in Saudi Arabia on March 2, 2020 [2], the Ministry of Interior in Saudi Arabia took restricted orders immediately to limit and control the people's movement inside the country and prevent transport in and out of some cities, started a partial curfew from 7 pm to 6 am on March 23, 2020, all over the cities [3]. A 24-hour curfew was announced in Taif City on April 6 [4]. Movement was completely restricted except for necessary services and emergency cases. The hospitals continued to receive and treat emergency trauma cases, and because of limited movement, the number of trauma patients decreased. This demonstrated the impact of the COVID-19 pandemic on the epidemiology of fractures.

A study done in Al-Khobar City showed that the lockdown period during the COVID-19 pandemic resulted in a decrease in orthopedic trauma admissions [5]. In Berlin, Germany, a retrospective study was done for orthopedic trauma cases during the COVID-19 lockdown period (35 days) in comparison with the control group from the period in 2019, and the result showed a decreased admissions rate during the shutdown period compared to the CTRL period [6].

This study intended to highlight the change in the pattern and number of cases presented with fractures and show the management and admission rate at three main governmental hospitals in Taif City, Saudi Arabia, during the curfew period.

## Materials and methods

A retrospective study was performed in Alhada Armed Forces Hospital, King Abdulaziz Specialist Hospital, and King Faisal Medical Complex, Taif City, Saudi Arabia, between July and December 2023. The study sample consisted of all patients with fractures who came to the emergency room and were treated by the orthopedic department, whether conservatively or admitted for operation during the lockdown period from March 23, 2020, to June 21, 2020, and from March 23, 2019, to June 21, 2019. The two groups from the two different years were compared. The study sample consisted of 253 patients. The sample is considered a convenient non-probability sample. The inclusion criteria included all patients with fractures who arrived at the emergency room directly or were transported from another regional medical facility. The exclusion criteria included patients who came for follow-up regarding old fractures before the lockdown period. Data were collected from patient's medical records. The data included the patient's age, gender, fracture type, fracture site, the mechanism of injury, whether the patients were admitted or not, length of hospital stay if admitted, and the plan of management.

Data analysis

Following data extraction, it was edited, coded, and entered into IBM SPSS version 22, a statistical program (SPSS, Inc. Chicago, IL). Two-tailed tests were used for all statistical analyses. P-values below 0.05 were considered statistically significant. Descriptive analysis using the frequency and percent distribution was conducted for each category variable. (personal data, pattern of fracture, and management).

Meanwhile, a mean with standard deviation was used to display numeric variables (hospital stay). Crosstabulation was used to compare all data, including personal characteristics, fracture features, and management, between patients before and during the COVID-19 pandemic periods. The relations were tested using Pearson's chi-square and exact probability test for small frequencies.

This study received approval from the Research Ethics Committee of Armed Forces Hospitals (Approval No. 2023-739) and the Directorate of Health Affairs, Taif Research and Studies Department (Approval No. 790).

## Results

One hundred eighty-four patients with fractures attended the emergency room before the COVID-19 pandemic (group 1), and 69 patients with fractures attended the ER during the COVID-19 pandemic lockdown (group 2). They were compared by age and gender with no statistically significant difference (p=0.140 and 0.607, respectively) (Table 1).

**Table 1:** Personal characteristics of patients with fractures who came to the ER before and during the COVID-19 pandemic, Taif City, Saudi Arabia P: Pearson X2 test; $: Exact probability test; N: Number

Personal data	Phase				P-value
	Pre-COVID-19 pandemic (n=184)		During COVID-19 pandemic (n=69)		
	N	%	N	%	
Age in years					0.140^$^
< 17	64	34.8%	21	30.4%	
17-30	47	25.5%	25	36.2%	
31-45	37	20.1%	7	10.1%	
> 45	36	19.6%	16	23.2%	
Gender					0.607
Male	134	72.8%	48	69.6%	
Female	50	27.2%	21	30.4%	

The pattern of fractures among patients before versus during the COVID-19 pandemic in Taif, Saudi Arabia, was compared. The fracture type was closed fractures among 88.6% of group 1 patients compared to 97.1% of group 2 patients with recorded statistical significance (P=0.036). Regarding site of fracture, the most reported sites among group 1 patients were foot fractures (18.5%), finger fractures (15.2%), femur fractures (13%), radius/ulna fractures (12%), and tibia/fibula fractures (12%). Among group 2 patients, the most reported fracture sites included fingers (21.7%), femur (14.5%), foot (11.6%), toes (10.1%), humerus (8.7%), tibia/fibula (8.7%), and distal radius (8.7%). A significant difference was detected for radius/ulna (P=0.035) and foot fractures (0.010). Regarding the mechanism of injury, the most reported mechanism was a simple fall, 39.1% for group 1 vs. 49.3% for group 2. A significant difference was reported for simple falls (P=0.032), MVA (0.009), and other trauma (P=0.042) (Table 2).

**Table 2:** Comparison of the features of fractures among trauma patients before versus during the COVID-19 pandemic, Taif City, Saudi Arabia P: Exact probability test; * P < 0.05 (significant); N: Number

	Phase				
Fracture data	Pre-covid-19 pandemic (n=184)		During-covid-19 pandemic (n=69)		P-value
	N	%	N	%	
Type of fracture				0.036*	
Closed fracture	163	88.60%	67	97.10%	
Open fracture	21	11.40%	2	2.90%	
Site of fracture					
Shoulder	4	2.20%	2	2.90%	0.421
Supracondylar humerus	12	6.50%	2	2.90%	0.086
Humerus	11	6.00%	6	8.70%	0.091
Radius/Ulna	22	12.00%	4	5.80%	0.035*
Elbow	1	0.50%	0	0.00%	0.884
Hand	10	5.40%	5	7.20%	0.254
Fingers	28	15.20%	15	21.70%	0.214
Hip	2	1.10%	1	1.40%	0.743
Femur	24	13.00%	10	14.50%	0.067
Tibia/ fibula	22	12.00%	6	8.70%	0.084
Patella	4	2.20%	0	0.00%	0.716
Distal radius	11	6.00%	6	8.70%	0.091
Foot	34	18.50%	8	11.60%	0.010*
Toes	5	2.70%	7	10.10%	0.694
Mechanism of injury					
Simple fall	72	39.10%	34	49.30%	0.032*
Fall from height	7	3.80%	1	1.40%	0.407
Gunshot injury	1	0.50%	0	0.00%	0.896
Human bite	1	0.50%	0	0.00%	0.896
Machine injury	1	0.50%	0	0.00%	0.896
Motor vehicle accident	41	22.30%	7	10.10%	0.009*
Sport injury	1	0.50%	0	0.00%	0.896
Twisted	1	0.50%	3	4.30%	0.705
Other trauma	59	32.10%	24	34.80%	0.042*

As for the management plan, 47.8% of group 1 patients underwent operative management compared to 31.9% of group 2 patients, while conservative treatment was needed for 50.5% of group 1 versus 66.7% of group 2 patients with recorded statistical significance (P=0.049). A total of 65.8% of group 1 patients were hospitalized compared to 33.3% of group 2 patients (P=0.001). Hospitalization average days were 4.2 ± 3.7 for patients before covid-19 pandemic versus 3.7 ± 2.0 days for patients during covid-19 lockdown (P=0.501) (Table 3).

**Table 3:** Comparison of management and hospitalization of fractures among trauma patients before versus during the COVID-19 pandemic, Taif, Saudi Arabia P: Exact probability test; * P < 0.05 (significant); N: Number

Management/hospitalization	Phase				P-value
	Pre-covid-19 pandemic (n=184)		During-covid-19 pandemic (n=69)		
	N	%	N	%	
Plan of management					0.049*
Operative	88	47.8%	22	31.9%	
Conservative	93	50.5%	46	66.7%	
Discharge against medical advice	3	1.6%	1	1.4%	
Hospital admission					0.001*
Yes	121	65.8%	23	33.3%	
No	63	34.2%	46	66.7%	
Duration of stay in days					0.501
1-2 days	45	37.2%	6	26.1%	
3-6 days	53	43.8%	13	56.5%	
7+ days	23	19.0%	4	17.4%	
Mean ± SD	4.2 ± 3.7		3.7 ± 2.0		

## Discussion

In this study, we describe the change in the pattern of fractures during the COVID-19 pandemic in Taif City in March-June 2019 and 2020.

Overall, 184 trauma cases attended ER before the COVID-19 pandemic before the COVID-19 pandemic and decreased during lockdown by 62.5% with no statistical significance. In Al-Khobar City, Saudi Arabia, the University Hospital found that there were 71 admissions during the quarantine compared to 110 in the previous year. The patient's number reduced by 35.4%, mainly in women and children groups [5]. The study showed no significant association between demographics of trauma cases before and during the COVID-19 pandemic, and this is logical as personal characteristics for susceptible personnel to trauma will not be affected by the trauma phase (nearly the same age and gender). Similar findings were found in the Al-Khobar study regarding age and gender differences, where the average age and adults' gender were similar before and during the COVID-19 pandemic [5]. In contrast, a study done in Pennsylvania reported significant differences in traumatized ages but not for gender [7].

As for features epidemiologically, the current study showed that closed fractures were significantly higher during COVID-19 than open fractures. This may be explained by the fact that most fractures were low-energy fractures due to lockdown and quarantine, which are mostly less severe than high-energy fractures, which are usually associated with external injury. The most reported fracture sites higher during the COVID-19 pandemic included femur and humerus fractures, besides finger fractures (which may be associated with falls due to excessive in-home movement with lockdown), but failed to show statistical significance. This can be proven by the fact that simple falls as a trauma mechanism were significantly higher during the COVID-19 pandemic, but motor vehicle accidents were lower than in the pre-COVID phase. Other types of trauma, mainly foot fractures and radius /Ulna fractures, significantly decreased during the COVID-19 pandemic. No clear explanation can be reported, but the differences in fracture rates are not clinically noticeable. Similar findings were also reported by a study in Al-Khobar City, Saudi Arabia, as most injuries were sustained from domestic and road traffic accidents. There was a significant increase in the vertebral column fractures (P < 0.002) and distal radius fractures (P < 0.05) [5]. Five of seven trauma centers in Pennsylvania have recently reported that there were fewer orthopedic injuries (4868 vs. 6603 yearly mean) in the COVID cohort, which led to fewer procedures (1763 vs. 2329 yearly mean [7].

Similarly, a tertiary care teaching hospital reported a sharp reduction in RTA-related orthopedic trauma in Pakistan during the COVID-19 pandemic [8]. Also, at a Southwestern Level-1 Trauma Center, the nature of injury mechanisms has changed [9]. Another study showed the pattern of pediatric injuries after two months of lockdown in France. The study showed that pediatric trauma was reduced by up to 50%. There were 59% more domestic accidents (compared to 23%) and 16% more trampoline accidents reported (compared to 5%) [10]. Likewise, a study in Madrid, Spain, reported that the trend of hip fractures decreased by up to 26% compared to the previous year before the pandemic [11].

Concerning changes in the management and hospitalization of fractures, the current study showed a reduction in surgical interventions, but more conservative interventions were needed. This is proportional to the nature and severity of reported fractures during the pandemic (closed fractures and less severe). Consequently, the need and duration of hospitalization were reduced during the pandemic. This was in accordance with a study done in Europe, which conducted a systematic review and revealed that many studies show a decrease in orthopedic consultations, which decreased between 20.9 and 90.1%. Seven studies evaluated the number of emergency and trauma consultations, which decreased between 37.7 and 74.2% [12]. Also, the Al-Khobar study reported that in the pre-COVID-19 period, the average discharge time was 18.90 ±12.74 days, and during the COVID-19 period, the average discharge time was 4.28 ± 3.52 days [5].

While this study shows the changes in the numbers and patterns of fractures during the COVID-19 pandemic and the impact of quarantine, it has a limitation centered on the fact that it is a retrospective study that depends on data from patient's records, which may contain incomplete or missing information, leading to potential bias.

## Conclusions

We concluded that the shutdown period during the COVID-19 pandemic did affect the pattern of fractures in Taif City, and the effect was close to our expectations. The number of fractures fell by approximately two-thirds during quarantine compared with the same period in 2019. The most common sites of fractures changed between pre-pandemic and shutdown periods: foot fractures and finger fractures. Demographic distribution did not show significant trends. The number of admitted patients and the duration of hospital stay decreased significantly during the quarantine period.
